# Genetic Evolutionary Analysis of Lumpy Skin Disease Virus Strain Under Immune Pressure Exerted by Heterologous Goat Poxvirus Vaccines

**DOI:** 10.1155/tbed/2883245

**Published:** 2025-02-23

**Authors:** Wenchi Chang, Junyang Fang, Tianshu Zhai, Shuizhong Han, Wenqi Fan, Congshang Lei, Lizhen Wang, Xuefeng Qi, Qinghong Xue, Jingyu Wang

**Affiliations:** ^1^Northwest A&F University College of Veterinary Medicine, Yangling, Shaanxi, China; ^2^China Institute of Veterinary Drug Control, Beijing, China

**Keywords:** genetic evolution, lumpy skin disease virus, recombination, virus isolation

## Abstract

Lumpy skin disease (LSD) is an important infectious disease that threatens the global cattle industry. Recently, LSD has occurred frequently in Asia. The attenuated goat poxvirus (GTPV) vaccine is widely used to prevent LSD in cattle in China; however, sporadic cases of LSD still occur in immunized cattle. This study aimed to investigate the genetic evolution of isolated LSD virus (LSDV) strain under GTPV heterologous vaccine immune pressure. We isolated a new strain of LSDV, named LSDV/China/SX/2023, from a cattle herd immunized with a GTPV-attenuated vaccine in Shaanxi Province, Northwest China, and conducted whole-genome sequencing and genetic evolution studies. There are several open reading frames (ORFs) differences between the isolated strain and the Chinese reference strains, among which truncated expression of the Kelch-like protein encoded by ORF 19 may affect its growth characteristics. Phylogenetic analysis revealed that the isolated strain is in the same evolutionary clade as previous isolates from China and Southeast Asia. RDP4 and Simplot simultaneously showed that all strains in this branch had the same 15 recombination events, and there was one recombination event associated with the GTPV. This study presents the recent genetic evolution of LSDV under GTPV vaccine immune pressure and discusses the viral recombination events that are thought to influence transmission which are critical for the control and purification of LSD.

## 1. Introduction

Lumpy skin disease (LSD) is an acute or subacute infectious disease in cattle caused by the LSD virus (LSDV), a member of the genus *Capripoxvirus* (CaPV) in the family *Poxviridae*. Different cattle breeds are susceptible to LSDV, and in certain cases, it can be transmitted across species, such as giraffes and Indian antelopes [1, 2]. The morbidity of LSD generally reaches 90% and the infected cattle developing a fever and presenting with skin, mucous membrane, organ nodules, and other clinical symptoms, and the mortality is less than 10% [3]. In countries and regions where LSD is prevalent, the export of susceptible animals and animal-derived products is strictly restricted by trading countries, which has a marked impact on the development of the local animal husbandry economy. The World Organization for Animal Health lists LSD as a notifiable animal disease [3], with China listing it as a Class II animal infectious disease.

LSD occurs throughout the year, with the LSDV mainly transmitted horizontally by ixodes, flies, Culicoides, *Aedes aegypti*, and other arthropods [4–7]. In summer, when mosquitoes breed, there are large outbreaks of LSD. The sporadic cases in other seasons mostly caused by indirect contact, semen, digestive tract, and overwintering transmission of LSDV [8–12].

LSD was first discovered in Zambia in 1929 and has since spread from Africa to the Middle East and Europe [13]. In 2015, LSDV spread to the Caucasus region of Russia, and after many LSD cases occurred in 2016, the government effectively controlled its spread through goat poxvirus (GTPV) vaccines [14]. However, after the use of an attenuated LSDV vaccine in the northern border region of Kazakhstan in 2017, a vaccine-like recombinant strain was isolated from the Russian region bordering Kazakhstan in the same year [13, 15]. In 2019, LSD was first detected in Xinjiang, China [16], followed by reports in Fujian, Guangzhou, inner Mongolia, Hong Kong, and other regions of China [17–20]. Concurrently, other Asian countries such as Vietnam, Thailand, and India have experienced LSD outbreaks [21–23]. From 2020 onwards, the circulating strains of LSDV in Russia, China, and Southeast Asia comprised a single lineage, represented by LSDV/GD 01/China/2020 [24].

LSDV, sheep poxvirus (SPPV), and GTPV constitute the CaPV genus of *Poxviridae*. CaPV is a double-stranded DNA virus with a genome size of 151 kbp; the central coding region contains 156 putative gene open reading frames (ORFs), and the terminal region of the genome has low homology with other poxviruses. The functions of these differential genes may be related to the virulence and host range of the virus [25]. Genomic homology among LSDV, SPPV, and GTPV is >97% [26]. Therefore, CaPVs are difficult to distinguish from each other serologically and can induce heterologous cross-protection [25].

Since 2020, China has adopted GTPV (AV41)-attenuated vaccine immunization for LSD prevention and control. The GTPV vaccine provides a certain degree of protection. However, LSD remains sporadic in immunized cattle. Exploring the genetic variation in LSDV under the immune pressure of the GTPV (AV41) vaccine is important for the formulation of prevention and control measures for LSD.

Northwest China is the primary breeding area for beef and dairy cows. Here, a LSDV strain was isolated from a cattle farm in Shaanxi Province, Northwest China, and the farm was previously immunized with attenuated GTPV (AV41) vaccine. To reveal the transmission and genetic evolution of isolated LSDV strain under heterologous vaccine immune pressure, the whole genome sequence of the virus was compared with those of CaPV strains recently reported in China and neighboring countries.

## 2. Materials and Methods

### 2.1. Cells and Reagents

Madin–Darby bovine kidney (MDBK) cells were obtained from the American Type Culture Collection (ATCC CCL-22). The cells were cultured and maintained in Dulbecco's Modified Eagle Medium (DMEM) supplemented with 10% Fetal Bovine Serum (FBS) and penicillin (100 IU/mL) and streptomycin (10 μg/mL) at 37°C and 5% CO_2_. Specific pathogen-free (SPF) 9-day-old chicken embryos were provided by Boehringer Ingelheim Viton (Beijing, China). Mouse polyclonal antibodies against the LSDV A27 protein were prepared in our laboratory.

### 2.2. Sample Collection and Processing

From a cattle farm in Shaanxi Province, Northwest China, the skin and tracheal mucosal nodule contents of dead cattle suspected of having LSD were collected aseptically using scalpels and tweezers, temporarily stored in sterile test tubes, and transported to a designated laboratory at a low temperature throughout the process. Using sterile scissors and tweezers, 0.2 g of diseased tissue were removed, and five times the volume of precooled PBS was added to disperse the tissue using a tissue grinder. The posttreatment samples were thrice frozen and thawed at −80°C to release the virus in the cells. Then, the supernatant was collected by centrifugation at 5000 rpm for 10 min and used for qPCR detection and virus isolation. Part of the supernatant was used for qPCR detection, and the other mixed 1:1 with a penicillin–streptomycin-mixed solution (100x) and incubated overnight at 4°C to completed the pretreatment before virus isolation.

### 2.3. qPCR Detection and Virus Isolation

For qPCR detection, the EasyPure viral DNA/RNA kit (TransGen Biotech, Beijing, China) was used to extract viral DNA from the collected supernatant, and the eluted viral DNA was stored at −20°C. Fluorescent quantitative primers and probe were designed in the LSDV ORF 007 region for qualitative sample detection (Table 1). Primers and probe were synthesized by Tsingke Biotechnology (Beijing, China). The HS Premix Probe qPCR Kit III was purchased from Accurate Biotechnology (Hunan, China). A two-step qPCR amplification procedure was used as follows: 95°C for 30 s followed 45 cycles of 95°C for 5s and 60°C for 30 s. Target gene amplification and signal acquisition were performed using a Bio-Rad CFX Connect (California, America) instrument.

MDBK cells were cultured to 80% confluence, incubated with supernatant treated with penicillin–streptomycin solution for 2 h. The supernatant was discarded and DMEM supplemented with 2% FBS was added to continue the incubation to wait for the appearance of obvious cytopathic effect (CPE). If no obvious CPE was observed, the cell samples were collected by repeated freeze-thawing for the next passaging before the cell state deteriorated, and the samples were passed through three to four generations until the CPE was stable. After 80% of the cells showed CPE, they were thrice frozen and thawed, centrifuged at 5000 rpm for 10 min to collect the supernatant, and refrigerated at −80°C for later use.

For the chicken embryo isolation virus, the treated samples were inoculated with 9-day-old SPF chicken embryos through a chorioallantoic membrane and cultured in an incubator at 38°C for 5 days. Chicken embryos that died within 24 h after inoculation were discarded, and the embryos were dissected after 5 days to observe any chorioallantoic membrane lesions. The parts with thickened chorioallantoic membranes and pockmarks were collected. After suspension in PBS, the supernatant was thrice frozen and thawed and centrifuged at 5000 rpm for 10 min. The collected samples were passaged three times in succession on new SPF chicken embryos.

### 2.4. Indirect Immunofluorescence Test

To determine whether the isolated virus bound to LSDV-specific antibodies, LSDV-infected MDBK cells were immobilized with 4% paraformaldehyde for 30 min and then infiltrated with 0.2% Triton X-100 for 20 min. A mouse polyclonal antibody against the LSDV A27 protein was used as the primary antibody at a dilution ratio of 1:500; FITC-labeled goat anti-mouse IgG (H + L; Biodragon, Jiangsu, China) was used as the secondary antibody, diluted at 1:1000; and DAPI solution (Solarbio, Beijing, China) was used to label the nucleus and perform an indirect immunofluorescence assay. The stained cells were observed, and images were collected using a Zeiss inverted fluorescence microscope.

### 2.5. Transmission Electron Microscope

MDBK cells with 80% confluence were inoculated with LSDV (MOI = 5) and incubated for 2 h. After replacement of the maintenance medium, the cells were cultured until notable CPE occurs. Trypsin was used to digest dispersed cells and 3% glutaraldehyde to fix the cells. Finally, JEM-1400FLASH transmission electron microscope (Japan Electronics Corporation) was used for image acquisition.

### 2.6. Virus Biological Characteristics

Virus titers of the LSDV/China/SX/2023 isolates were determined using the TCID_50_ assay and calculated using the Reed–Muench method. The LSDV growth curve was measured in MDBK cells which were inoculated at one MOI infection dose, and samples were collected every 24 h after inoculation for 9 days. Finally, viral titers at each time point were measured using TCID_50_.

### 2.7. Whole-Genome Sequencing Analysis

First, high quality viral DNA was prepared using the EasyPure viral DNA/RNA kit, then sequencing libraries were constructed using Covaris M220 fragment viral genomic DNA and the TruSeq DNA sample prep kit. Sequencing was performed according to the Illumina NovaSeq 6000 protocol, and the data were sorted and analyzed. SOAPdenovo (version 2.04) was used for the genome splicing and assembly, and the assembled viral genome was named LSDV/China/SX/2023.

### 2.8. Phylogenetic and Gene Recombination Analyses

Using the CaPV whole genome sequence in the NCBI database as a reference (Table 2), Mafft was used to perform multiple sequence alignments of 38 CaPV genome sequences, including LSDV/China/SX/2023. Trim and align sequences were performed with the multiple alignment trimming plugin in Tbtools. The maximum likelihood method in Mega 11 was used to construct an evolutionary tree to characterize the evolutionary and kinship relationships between LSDV/China/SX/2023 and the previously reported CaPVs (BOOTSTRAP value set to 1000). Itol (https://itol.embl.de/itol.cgi) was used to construct the evolutionary tree.

Based on the CaPV genome alignment sequence, the viral recombination event was analyzed using the RDP4 software and seven methods: RDP, GENECONV, Chimera, MaxChi, BootScan, SiScan, and 3Seq. Based on RDP4 analysis, recombination events were further verified and characterized using Simplot.

## 3. Results

### 3.1. Virus Isolation and Culture Characteristics

**Figure 1:**
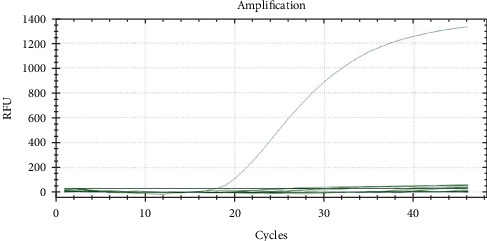
Fluorescence amplification curve of suspected lumpy skin disease virus (LSDV) infected sample identified by qPCR with CT value of 18.

**Figure 2:**
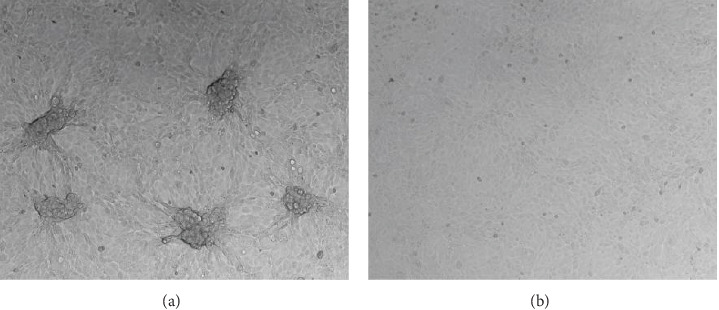
Typical cytopathic effect (CPE) after lumpy skin disease virus (LSDV) inoculation with Madin–Darby bovine kidney (MDBK) cells. (A) LSDV inoculated with MDBK cells group showed cytopathic lesions. (B) Normal MDBK cells control group.

**Figure 3:**
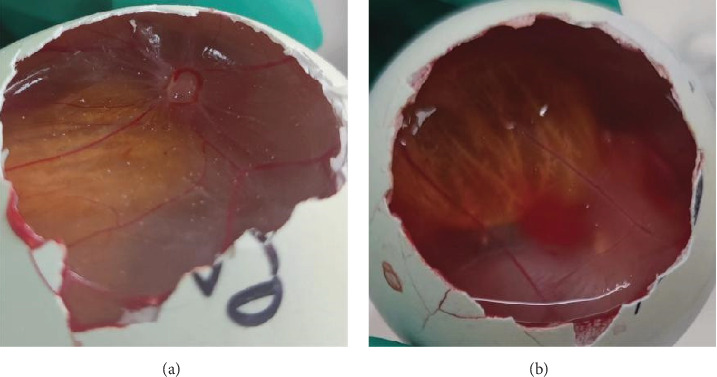
Membrane lesions of specific pathogen-free (SPF) chicken embryos after lumpy skin disease virus (LSDV) inoculation through a chorioallantoic membrane. (A) Chorioallantoic membrane of LSDV-inoculated chicken embryos with marked thickened and pockmarks. (B) Normal chorioallantoic membrane of chicken embryos.

qPCR primers and probes targeting the 007 ORF region of the LSDV genome were used for qualitative detection of LSDV. The CT value of the tested samples was 18 and a negative control was established (Figure 1). After antibiotic treatment, the sample supernatant with positive qPCR results was inoculated into MDBK cells. CPE appeared in the cells after two generations of blind transmission, manifesting as local cells aggregation, and surrounding cells were deformed and elongated (Figure 2). The chorioallantoic membranes of SPF chicken embryos inoculated with the positive samples showed notable mucosal thickening and specific acne-like lesions (Figure 3).

### 3.2. Indirect Immunofluorescence Identification

**Figure 4:**
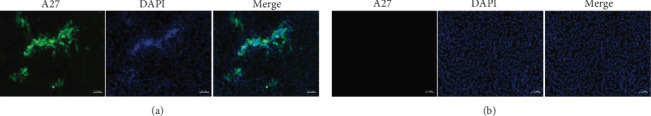
Indirect immunofluorescence analysis of Madin–Darby bovine kidney (MDBK) cells infected with isolated lumpy skin disease virus (LSDV) strain at 72 hpi. (A) Localized aggregated positive staining after inoculation of MDBK cells with LSDV. (B) Normal MDBK cells control group.

To determine whether local cell lesions were caused by LSDV-specific infection, an indirect immunofluorescence assay was performed using a preprepared anti-LSDV A27 protein mouse polyclonal antibody. Under a fluorescence electron microscope, specific green fluorescence was clearly observed at the site of clustered cytopathic lesions, and no green fluorescence was observed in the non-CPE and uninfected groups (Figure 4).

### 3.3. Observation by Transmission Electron Microscope

**Figure 5:**
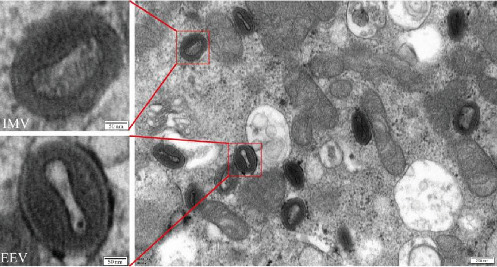
Transmission electron microscopy field of view of intracellular lumpy skin disease virus (LSDV) particles. The diameter of the brick- or oval-shaped viral particles is around 200–250 nm.

Poxvirus virions are generally >200 nm in diameter; most of them are brick- or oval-shaped and have two forms: extracellular enveloped virions (EEVs) and intracellular mature virions (IMVs) [27]. The LSDV-infected cells were fixed with glutaraldehyde and observed under a transmission electron microscope. Many uniform particle size virions in the range of 200–250 nm were observed. The particles were either brick or oval shaped. In the same field of view, two viral morphologies were observed, EEVs and IMVs, in which EEVs clearly had a double envelope morphology (Figure 5).

### 3.4. Virus Biological Characteristics

**Figure 6:**
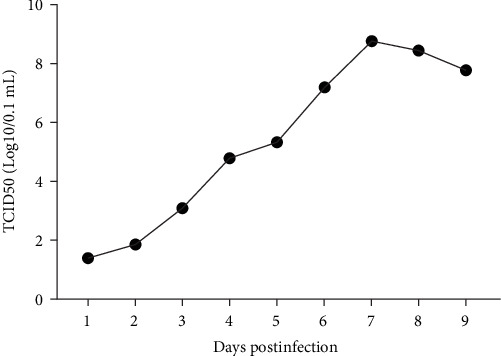
Growth curve of lumpy skin disease virus (LSDV)/China/SX/2023 on Madin–Darby bovine kidney (MDBK) cells.

The TCID_50_ of LSDV used to perform the virus biological characteristics experiment was measured to be 10^−5.8^/mL. After inoculation of MDBK cells with LSDV at one MOI infection volume, the viral titer gradually increased from the first day after inoculation, peaked on the 7th, and began to slowly decline thereafter (Figure 6).

### 3.5. Genome Sequence Comparison Between LSDV Isolates and Reference Strains

We uploaded the whole genome sequence of the isolated LSDV strain to NCBI database and named it LSDV/China/SX/2023 (GenBank: PP894832). The nucleotide identities of the LSDV/China/SX/2023 and LSDV strains isolated in China were >99.9%. The nucleotide homogeneity of LSDV/China/SX/2023 with GTPV and SPPV was <98.0%, whereas that of LSDV/China/SX/2023 with GTPV AV41 was only 97.6% (Table 3).

A sequence comparison revealed that LSDV/China/SX/2023 had base insertions or mutations at ORFs 19, 59, and 143 compared with LSDV/China/XJ01/2019, which directly led to changes in the amino acid homology of the Kelch-like protein (ORF 19), poxvirus myristoyl protein (ORF 59), and tyrosine protein kinase-like protein (ORF 143) with altered amino acid homology (Table 4). Compared with LSDV/China/GD01/2020, LSDV/China/SX/2023 had five ORFs coding for products with <100% identity, namely, the hypothetical protein (ORF 1), Kelch-like protein (ORF 19, ORF 144), putative ER-localized apoptosis regulator (LSDV154), and hypothetical protein (LSDV155; Table 4). LSDV/China/SX/2023 showed no correlation between the base insertions or mutations in these ORFs and their corresponding positions in GTPV and SPPV.

**Figure 7:**
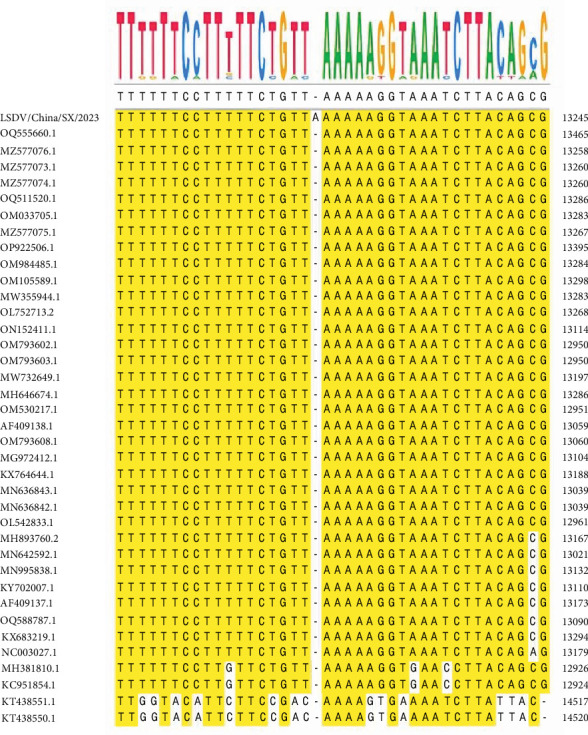
The presence of a single base A insertion at position 13229 in the lumpy skin disease virus (LSDV)/China/SX/2023 viral genome, a mutation that is unique.

Notably, the LSDV/China/SX/2023 viral genome contains a base A insertion at 13229. This base insertion causes the length of ORF 19 change from 1323 to 873 bp, and the molecular weight of the Kelch-like protein encoded by it change from 51.4 to 33.5 kDa. In addition, the base insertion was specific to the whole genome sequence of 37 CaPV strains worldwide and was not found in either vaccine or field strains (Figure 7).

### 3.6. Evolutionary Tree Analysis of LSDV Isolates

**Figure 8:**
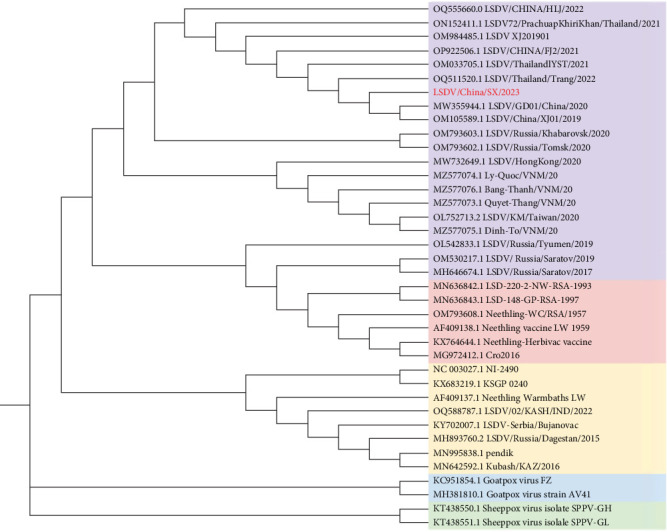
Maximum likelihood phylogenetic tree characterizing the genetic relationships of 38 *Capripoxvirus* (Capv) complete genome sequences. New lumpy skin disease virus (LSDV) strain are highlighted in red font, and LSDV recombinant, LSDV vaccine, LSDV wild, goat poxvirus (GTPV), and sheep poxvirus (SPPV) strains clades are labelled in purple, pink, yellow, blue and green, respectively.

Phylogenetic trees constructed using maximum likelihood suggest that LSDV/China/SX/2023 is in the same clade as the LSDV recombination strains isolated from China and Southeast Asia after 2019, as well as the two LSDV strains isolated from Russia after 2020. LSDV phylogeny can be divided into three main branches of vaccine, field, and vaccine-recombinant strains, and there are three secondary branches of vaccine-recombinant strains, the most important of which is composed of the Asian-recombinant strains (Figure 8). SPPV and GTPV are two separate evolutionary clades genetically closer to the LSDV field strains.

### 3.7. Virus Recombination Event Prediction

**Figure 9:**
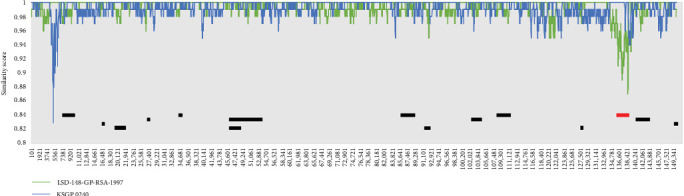
RDP4 and Simplot analyses of recombination events and positions in the whole genome of lumpy skin disease virus (LSDV)/China/SX/2023. The line graph depicts the genomic similarity of LSDV/China/SX/2023 versus LSD-148-GP-RSA-1997 (green) and KSGP 0240 (blue). The location and number of recombination events are labeled using the bar, where recombination event associated with goat poxvirus (GTPV) is highlighted in red.

To identify recombination events in the LSDV/China/SX/2023 strain, seven methods in RDP4 were used to predict the viral recombination events and sites. The prediction results showed that there were 15 recombination events in LSDV/China/SX/2023 with each event supported by at least two methods (*p*  < 0.1). LSDV/China/SX/2023 had the same recombination event as LSDV strains isolated from China and Southeast Asia after 2019. To predict recombination event of CaPV, we jointly analyzed the genomic data for GTPV. Notably, 17 recombinant strains from Asia had a GTPV gene recombination event in the breakpoint region (135621–138634), one of the 15 recombination events. The Simplot software and RDP4 results corroborated (Figure 9).

## 4. Discussion

LSDV was first discovered in Africa, and along with economic development and commodity trade, it gradually spread to the Middle East and Europe [13]. The 2019 outbreak in Yili Prefecture, Xinjiang Province, China confirmed the first introduction of LSDV into China [16]. At present, China, Russia, and Southeast Asian countries have not adopted LSDV-attenuated vaccine strains for the prevention and control of this disease; instead, LSDV-inactivated or GTPV-attenuated vaccines are used. Recently, there have been continuous outbreaks of LSDV in China and neighboring countries, and a single lineage represented by LSDV/China/GD01/2020 has been formed [24]. The effect of immune stress under GTPV (AV41)-attenuated vaccine immunoprophylaxis on genetic variation in sporadic LSDV strains remains unclear.

Here, we isolated an LSDV strain from a herd immunized with a GTPV (AV41) hetero-attenuated vaccine and named it LSDV/China/SX/2023. The whole genome virus was sequenced in the second generation and the whole genome sequence was successfully obtained and uploaded to the NCBI database.

CaPV is a double-stranded DNA virus with a highly conserved genome. The genomic homology between LSDVs was >99% and that between LSDV, GTPV, and SPPV was 97–98%. The whole genome sequence of LSDV/China/SX/2023 was compared with that of the reference strains (LSDV/China/XJ01/2019 and LSDV/China/GD01/2020), and several gene mutations and insertions were found, resulting in differential changes in the coding products of multiple ORFs. This may be a normal genetic variation in LSDV strains in the process of transmission and evolution; however, the role of GTPV-attenuated vaccine immune pressure cannot be ignored.

The most notable alteration of ORFs in LSDV/China/SX/2023 was a single A-base insertion event in ORF 19, resulting in a notably truncated expression of the encoded Kelch-like protein. CaPV contains three Kelch-like proteins encoded by ORF 19, 144, and 151 [25, 26]. ORF 19 is known to be associated with virulence of the virus. ORF 19-deficient and maternal SPPV strains were inoculated into sheep, and the gene deletion strain showed lower pathogenicity and virus shedding [28]. Studies have reported that Kelch-like proteins control the assembly and depolymerization of actin microfilaments, participate in the regulation of cytoskeletal morphology and the formation of pseudopods, and regulate cytoplasmic flow and communication between cells [29]. During the process of vaccinia virus MV invasion, the virions bind with the filopodia of the cell and move towards the cell body, and when they approach the cell plasma membrane, they enter the cell via giant pinocytosis [30]. However, whether the alteration of the Kelch-like protein (ORF 19) in LSDV/China/SX/2023 affects virulence, and the process of virus entry, require further investigation. The highest viral titer was observed in MDBK cells infected with LSDV at 5 dpi, and on the 3rd day after primary bovine testicular cells were infected [31]. We measured the growth curve of LSDV/China/SX/2023 in infected MDBK cells. The viral titer peaked at 7 day after exposure, followed by a gradual decrease along with cell shedding. It is possible that the truncated expression of the Kelch-like protein (ORF 19) affects the timing of the viral growth curve peak.

The phylogenetic tree analysis showed that from the first LSDV case in China in 2019 to the widespread outbreak in Southeast Asia, the region formed a unique branch of recombinant viruses in a short period. The LSDV recombinant strains isolated in Russia in 2017 and 2019 belonged to a unique recombinant branch, but the two LSDV recombinant strains isolated in 2020 belonged to the same branch as recombinant viruses in China and Southeast Asia. The decreasing evolutionary distance of LSDV in neighboring countries is closely related to the increasing frequency of communication in various fields. The results of the recombination event prediction showed that after 2019, the LSDV recombination strains in China and neighboring Southeast Asian countries had the same 15 recombination events. Notably, one GTPV recombination event was observed in 17 LSDV-circulating strains in Asia. Here, evidence of recombination between CaPV strains has been presented, which has not been reported in previous studies.

After the introduction of the attenuated LSDV vaccine in Kazakhstan and other Asian regions in 2017, strains recently circulating in Asia have shown recombination characteristics of vaccine and field strains. After the introduction of LSDV in China, the currently isolated strains showed recombination characteristics [18, 20, 32]. However, it is unknown whether the existing recombinant strains will undergo a new recombination phenomenon with heterogeneous GTPV-attenuated strains used for immunoprophylaxis in the process of genetic evolution. No new recombination events were observed in the LSDV/China/SX/2023 strain. The genome homology between CaPV strains is very high, and although it has cross-protection abilities, the protection rate is still insufficient [33, 34]. In the present study, the confirmation of a case of LSDV in a herd immunized with the attenuated strain of GTPV AV41 further demonstrates the inadequacy of existing immunization measures. In the long run, LSDV infection after GTPV-attenuated strain immunization may also occur, and whether new LSDV recombinant strains will emerge after mixed infection requires long-term continuous monitoring.

Viral recombination events have different degrees of influence on viral resistance, modes of transmission, and virulence. The high level of genomic similarity between multiple recombinant viruses isolated over time in East and Southeast Asia is evidence of a gradual increase in viral transmission across the region. It is inevitable that recombinant strains in East Asia and Southeast Asia will spread to neighboring countries and regions, and possible subsequent new recombinations will pose serious challenges for the prevention and control of LSDV globally. Therefore, a complete understanding of the genetic evolution and gene recombination of LSDV is essential for subsequent research on vaccine design, antiviral therapy, and monitoring and control of virus transmission.

## 5. Conclusion

The northwest region of China is one of the main concentration areas of the cattle industry, and LSD is a major disease that harms this industry. Here, we isolated and identified the LSDV/China/SX/2023 strain for the first time in Shaanxi Province, Northwest China. Importantly, the LSDV/China/SX/2023 strain were derived from cattle immunized with the attenuated GTPV (AV41) vaccine. Whole genome sequence analysis is of high importance for understanding the evolution of LSDV during transmission and provides new insights into the evolutionary model of LSDV.

## Figures and Tables

**Table 1:** Fluorescent quantification of primers and probe.

Primers and probes	Sequence (5′→3′)
007-F	AATCGTTTTTTGATTTTGGATACCA
007-R	TTGATTACTTAAAAAACGAAGATGGAAA
007-P	5′FAM-ATCACACGACGAATTTAAGGCT-3′MGB

**Table 2:** Information of 37 CaPV strains for LSDV whole genome sequence comparison and analysis.

Country	Type	Year	Name	GenBank ID
China	Recombinant	2020	LSDV/GD01/China/2020	MW355944.1
	Recombinant	2019	LSDV XJ201901	OM984485.1
	Recombinant	2019	LSDV/China/XJ01/2019	OM105589.1
	Recombinant	2022	LSDV/CHINA/HLJ/2022	OQ555660.1
	Recombinant	2021	LSDV/CHINA/FJ2/2021	OP922506.1
	Recombinant	2020	LSDV/HongKong/2020	MW732649.1
	Recombinant	2020	LSDV/KM/Taiwan/2020	OL752713.2

Russia	Recombinant	2017	LSDV/Russia/Saratov/2017	MH646674.1
	Recombinant	2019	LSDV/Russia/Saratov/2019	OM530217.1
	Wild-type	2015	LSDV/Russia/Dagestan/2015	MH893760.2
	Recombinant	2020	LSDV/Russia/Tomsk/2020	OM793602.1
	Recombinant	2020	LSDV/Russia/Khabarovsk/2020	OM793603.1
	Recombinant	2019	LSDV/Russia/Tyumen/2019	OL542833.1

Kazakhstan	Wild-type	2016	Kubash/KAZ/2016	MN642592.1

Vietnam	Recombinant	2020	Bang-Thanh/VNM/20	MZ577076.1
	Recombinant	2020	Quyet-Thang/VNM/20	MZ577073.1
	Recombinant	2020	Dinh-To/VNM/20	MZ577075.1
	Recombinant	2020	Ly-Quoc/VNM/20	MZ577074.1

India	Wild-type	2022	LSDV/02/KASH/IND/2022	OQ588787.1

Thailand	Recombinant	2021	LSDV72/PrachuapKhiriKhan/Thailand/2021	ON152411.1
	Recombinant	2022	LSDV/Thailand/Trang/2022	OQ511520.1
	Recombinant	2021	LSDV/Thailand/YST/2021	OM033705.1

South Africa	Vaccine	1959	Neethling vaccine LW 1959	AF409138.1
	Vaccine	1957	Neethling-WC/RSA/1957	OM793608.1
	Vaccine	1997	LSD-148-GP-RSA-1997	MN636843.1
	Vaccine	1993	LSD-220-2-NW-RSA-1993	MN636842.1
	Wild-type	1999	Neethling Warmbaths LW	AF409137.1

Kenya	Wild-type	2016	KSGP 0240	KX683219.1
	Wild-type	2001	NI-2490	NC_003027.1

Croatia	Vaccine	2018	Cro2016	MG972412.1

Turkey	Wild-type	2020	pendik	MN995838.1

Belgium	Vaccine	2016	Neethling-Herbivac vaccine	KX764644.1

Serbia	Wild-type	2017	LSDV-Serbia/Bujanovac	KY702007.1

China	Vaccine	2019	Goatpox virus strain AV41	MH381810.1
	Wild-type	2014	Goatpox virus FZ	KC951854.1
	Wild-type	2017	Sheeppox virus isolate SPPV-GL	KT438551.1
	Wild-type	2017	Sheeppox virus isolate SPPV-GH	KT438550.1

Abbreviations: CaPV, *Capripoxvirus*; LSD, lumpy skin disease; LSDV, LSD virus; SPPV, sheep poxvirus.

**Table 3:** Comparison of genomic identity between LSDV/China/SX/2023 and CaPV strains isolated from China.

CaPV strains	Identity compared to LSDV/China/SX/2023 (%)
LSDV/GD01/China/2020	99.96
LSDV XJ201901	99.96
LSDV/China/XJ01/2019	99.97
LSDV/CHINA/HLJ/2022	99.97
LSDV/CHINA/FJ2/2021	99.97
LSDV/HongKong/2020	99.93
LSDV/KM/Taiwan/2020	99.98
Goatpox virus strain AV41	97.60
Goatpox virus FZ	97.97
Sheeppox virus isolate SPPV-GL	97.87
Sheeppox virus isolate SPPV-GH	97.87

Abbreviations: CaPV, *Capripoxvirus*; LSD, lumpy skin disease; LSDV, LSD virus; SPPV, sheep poxvirus.

**Table 4:** ORFs for nonidentity of LSDV/China/SX/2023 compared to reference strains against (China/XJ01/2019, China/GD01/2020).

Matched strain	ORFs	China/XJ01/2019 (%)	China/GD01/2020 (%)
LSDV/China/SX/2023	LSDV001	100	99.4
	LSDV019	65.9	65.9
	LSDV059	99.7	100
	LSDV143	97.1	100
	LSDV144	100	99.3
	LSDV154	100	99.6
	LSDV155	100	97.7

Abbreviations: LSD, lumpy skin disease; LSDV, LSD virus.

## Data Availability

The data that support the findings of this study are available from the corresponding author upon reasonable request.
